# Effects of Knowledge Hiding in Dual Teaching Methods on Students’ Performance—Evidence From Physical Education Department Students

**DOI:** 10.3389/fpsyg.2022.833285

**Published:** 2022-02-28

**Authors:** Qingxiang Xu, Yin Jiesen

**Affiliations:** ^1^PE Department, Jiangnan University, Wuxi, China; ^2^Department of PE, Wuxi Institute Technology, Wuxi, China; ^3^Graduate School Division José Rizal University, Mandaluyong, Philippines

**Keywords:** rationalized knowledge hiding, online teaching, physical teaching, students’ disengagement, students’ performance, academics

## Abstract

With the post-pandemic situation, digitalization has revolutionized physical teaching into online teaching and has become a common practice. The engagement of students has been essential for their good academic performance which can be ensured by the active participation of the students and this is a real challenge for the teachers. However, sometimes in online and physical teaching, teachers are also involved in rationalized knowledge hiding, which leads to the disengagement of the students, and this ultimately affects their academic performance. Therefore, the present study aims at measuring the students’ disengagement in the teaching classes, both physical and online. The population of the present study is the students from the universities of China belonging to different fields of study. The sample size for this study is 246. The data are obtained through the Questionnaire surveys. The existing study has assessed the role of teachers’ rationalized knowledge hiding behaviors in the disengagement of students and their lesser grades. It has been found that rationalized knowledge hiding in online teaching does not affect students’ performance; however, it makes students disengage from their studies in physical classes. Interestingly, the rationalized knowledge hiding in physical teaching has negatively affected the performance of the students. Furthermore, the mediating role of the students’ disengagement has been found significant in this study. Organizations, especially universities, can ensure maximum knowledge sharing by motivating the instructors through positive reinforcements. This study will be useful for the curriculum coordinators of different departments in ensuring the maximum outcome of the teaching classes, workshops, and seminars conducted either physically or online to avoid the rationalized knowledge hiding of the teachers.

## Introduction

Knowledge sharing and knowledge hiding during COVID-19 and after the lockdown across the globe have given a new dimension to different perspectives of research on this issue. Knowledge hiding in the teaching perspective has different types, having certain effects on the performance of the students. Many disciplines of organizational study on the function of knowledge and related behaviors have arisen as a result of early research on management and learning orientation (Creed and Zutshi, 2008). Knowledge may be divided into two categories in various organizations: tacit knowledge and explicit knowledge (Dampney et al., 2007). Knowledge may be divided into explicit information, which is more readily articulated and taught, and tacit knowledge, which is difficult to transmit since it is typically ingrained in identities, culture, and artifacts (Nonaka, 2009). Researchers went on to recognize that in people’s connections inside the companies, there are surface and deeper realms of social reality. Certain knowledge regarding the individuals and procedures in universities, such as complex database system operations or ingrained political contacts, is hidden and enigmatic, while other knowledge, such as organizational charts, policies, biographies, and research outputs, is apparent and understandable. As conditions change, these knowledge states fluctuate as well. Depending on the context and circumstances, what is hidden today may be revealed tomorrow (Hernaus et al., 2019).

Knowledge management research has mostly concentrated on the transfer of knowledge and knowledge sharing throughout the last few decades. Management researchers are more interested in knowledge hiding. This is unexpected, given the fact that many businesses have ongoing issues due to benign or malignant communication impediments (Garcia Martinez et al., 2019). Knowledge hiding is being distinguished from other similar but distinct notions like knowledge sharing and knowledge hoarding. Despite advances, few studies have looked at the topic of knowledge hiding in higher learning institutions, such as universities. Researchers have focused on the antecedents of knowledge hiding as one of the few exceptions (Silva de Garcia et al., 2020; Xiong et al., 2021). Playing dumb, evasive hiding, and rationalized hiding are the three different approaches/strategies for knowledge hiding. Rationalized hiding is an alternatively used term for justification-based knowledge hiding. When a person plays dumb, he or she pretends to be unaware of the information sought by the knowledge seeker. Even if the knowledge hider intends to conceal the knowledge, evasive hiding entails that the knowledge hider seems to reveal the knowledge to the seeker. Pretending may be done in a variety of ways, including presenting inaccurate knowledge/information or making a false promise to transfer the knowledge.

The third form, the rationalized hiding, is distinct from the other two. It entails the knowledge hider explaining why he or she is concealing the knowledge. Researchers discovered that those who were subjected to evasive hiding and playing dumb felt a sense of betrayal and, to some extent, a desire for revenge by withholding information from that person in the future. For the rationalized hiding strategy, this result could not be reached (Connelly and Zweig, 2014; Connelly et al., 2019). However, none of the prior studies looked at the link between the elements of knowledge hiding, such as evasive hiding, playing dumb and rationalized hiding, or rationalized knowledge hiding. Each aspect of knowledge hiding has a unique scenario, and these scenarios might have varying effects (both good and bad) on the knowledge seekers. For example, evasive knowledge hiding and playing dumb are both based on deceit; but a rationalized knowledge hider explains his role and justifies his knowledge hiding. The knowledge seekers may react poorly to evasive hiding and playing dumb behaviors in this scenario, but positively to reasoned knowledge hiding (Fong et al., 2018). Knowledge sharing has been proved to improve the learners’ teamwork, motivation, and satisfaction in the past. Nevertheless, few research studies have looked at why students are often hesitant to share or even hide their knowledge. In many cases, such conduct is classified as a knowledge-hiding problem, which can lead to intellectual enervation, negative dependency, and creativity blockage.

The knowledge that is hidden in disciplines, such as organizational behavior and knowledge management is receiving more attention (Lin et al., 2016; Al-Husseini and Elbeltagi, 2018). However, no similar study has been conducted in the education sector. Graduates of higher education make up a significant portion of individuals who will contribute to information exchange and transmission in the knowledge society (Välimaa and Hoffman, 2008). Since knowledge hiding in higher education deserves to be investigated, the fact that it is rarely mentioned in the educational sector might be related to the naive idea that expanding information sharing will solve the problem (Peng, 2013). Knowledge hiding, on the other hand, is not merely the absence of knowledge sharing or the polar opposite of knowledge sharing and both can occur at the same time. Knowledge sharing, for example, is usually associated with lessening the competition and establishing the shared benefits, whereas knowledge hiding can occur with or without competition, regardless of the shared advantages and values. Furthermore, while information sharing and knowledge hiding might appear in identical ways, they can also appear in opposite ways (Connelly and Zweig, 2014). As a result, it is critical to look at the dimensions and the methods of knowledge hiding behaviors in teaching in order to reduce them in the current and future leaders-students performance. Online and distant education have arisen in recent years as a convenient mode of learning for busy or international students, as well as a highly productive chance to improve research and independent thinking abilities.

Some teachers have simply transported their conventional teaching approaches to the online mode during COVID-19 owing to a lack of online teaching preparation, which may negatively affect students’ online learning and reduce their online class participation. Sometimes, this condition leads to knowledge hiding in teaching. Students get disengaged due to the reception of lesser knowledge from the teachers but the teachers owe to the justifications of such knowledge hiding in these circumstances. This thing ultimately affects the students’ performance in these kinds of situations. Nearly no research has been conducted on this aspect of students’ performance in the past which posed a gap in the research and allowed us to develop this dimension in the students’ performance. Knowledge hiding not only stifles students’ creativity, critical thinking, and collaborative learning, but also has a detrimental impact on the creation of a collaborative atmosphere, the establishment of shared academic materials, and interpersonal relationships (Littlejohn and Hood, 2017).

Student involvement is influenced by a variety of factors within and outside the classroom (Douglas and Alemanne, 2007). Academic behaviors, such as self-regulated learning techniques, mathematical reasoning, higher-order thinking activities, and reflective and integrative learning may all improve the content knowledge and the general cognitive processing. These are all connected to various elements of achievement and success. Student engagement also includes interactions with the teachers through student engagement and conversations with a variety of people, as well as student-faculty interactions and successful teaching approaches used by the teachers. Environmental factors, such as the quality of interactions with the students, instructors, and other forms of staff, as well as an overall sense of a supportive atmosphere, all contribute to student engagement (Baird, 2005). While many researchers have looked at the influence of pervasive digitalization on students’ education levels and academic development, little is known about how these alternative learning experiences and practices affect the overall student engagement, because most of the original research on student engagement focused on students in the traditional face-to-face settings (Junco, 2012; Dumford and Miller, 2018). In relation to the larger management environment, the study mentioned above provides some insights into the information that is hidden in higher education.

However, further research is needed to find how the characteristics of the higher education setting are connected to information hiding, as well as how to explain the academic knowledge hiding practices in terms of students’ disengagement from learning, which leads to poor performance. What, for example, is the rationalized information that is hidden in both online and physical learning, and what impact does it have on the students’ performance? How can a rationalized knowledge hiding result in student disengagement from the physical education system? What amount of flexibility is required in academic employment to strike a balance between information availability and knowledge hiding? Our research contributes significantly to the field of knowledge management. To begin with, we expand our knowledge on the phenomenon of knowledge hiding and the components that explain people’s knowledge-hiding intentions and behaviors in the context of education. This study focuses on the importance of rationalized types of knowledge hiding in academic contexts, instructors’ justification-based thinking, and their influence on the student involvement or disengagement in learning, as well as students’ performance. This research could provide the missing link between the impact of rationalized knowledge hiding on students’ performance and disengagement. This study could also provide information on the indirect effects of students’ disengagement on students’ performance.

## Review of Literature and Hypothesis Development

The model developed in our research refers to several social and management aspects, such as knowledge hiding in teaching, disengagement of the students, and the students’ performance. This research is based on the following theories of performance management, social exchange, and learning theories.

### Performance Management Theories

Performance management has traditionally been characterized as the management’s planned use of methods aimed at improving the performance level in a company. Performance targets, which trigger behavioral and direct outputs, a performance feedback loop for monitoring activities and outputs, and a formal performance evaluation phase are all added to this basic performance system (Goshu and Kitaw, 2016). The researchers’ list of characteristics that influence their imagined performance system raises six significant concerns for the management: (a) specificity of a performance; (b) support for the task; (c) consequences matching with the planned performance; (d) provision for the feedback; (e) prevalence of necessary skills and knowledge; and (f) the capacity to perform (MacLeod and Best, 2015).

In educational institutions, performance management is about developing and implementing the policies and procedures to make sure that the academic staff and teachers provide education and related services in such a way that effectively meet the requirements of the students and which are aligned with the objectives and goals of those institutions (Clandinin et al., 1996). Therefore, when in teaching processes, teachers rationally and logically hide any information or knowledge from their students, a deviation in the achievement of goals and objectives of the academic institutions is caused (Isherwood et al., 2007). Hence, performance management of the teachers and instructors should include the observation of teaching practices in the classrooms and online classes to gather useful information that could give room for improvement in the learning and teaching methodologies for both the students and teachers. So, these cases and theories related to performance management in the light of online and physical education provide the basis for our research.

### Social Exchange Theory

The origins of social exchange theory may be traced all the way back to 1958. Homans proposed a paradigm based on a mix of behaviorism and fundamental economics. Other investigations enlarged the bounds of Homans’ key notions in the years that followed. The concept of social exchange theory is founded on the idea that a connection between two individuals is formed through a cost-benefit analysis. In other words, it is a measure for determining how much effort an individual puts out in a one-on-one interaction. The evaluation of a relationship’s pluses and minuses may yield data that may be used to identify whether someone is putting too much effort into a relationship. The idea is distinctive in that it does not always use emotional measurements to assess relationships. Its methodical methods, on the other hand, rely on mathematics and logic to assess the relationship balance (Cook et al., 2013). The cornerstone of social exchange theory is built on a set of key assumptions about human nature and relationship dynamics. Humans, according to the first premise, want to seek out rewards and avoid penalties. Another premise is that a person begins an encounter with the goal of maximizing profit at the lowest possible expense; the individual is motivated by, “What s in it for me?” A third assumption is that people analyze the profit and expense of doing something before doing it. Finally, the theory posits that people are aware that the pay-out will differ from one person to another, as well as from one person to another over time (Whitham, 2021). Several researchers have found the engagement or disengagement of the workers due to social exchange of behaviors (knowledge-hiding behavior in this case) and concluded that certain behaviors could lead to disengagement and adversely impacted performance (Yin, 2018).

Self-interest drives the social exchange theory, which adopts a person’s social reasoning. The social exchange theory assumes that people are self-centered, even though exchange behaviors appear toward being selfish. In other words, an individual will first assess the potential gains derived from connecting with one another. The social exchange will not occur if none of the two parties is able to obtain adequate benefits. As a result, students will strive to develop a mutually beneficial balance in the exchange relationships and sustain a long-term positive social exchange connection while studying. In a nutshell, the social exchange theory states that the purpose of human action is to maximize the benefits while minimizing the costs (Blau, 1964). Once, the student gets disengaged due to the rationalized knowledge hiding of the teacher, he would certainly not perform well and it would harm the repute of the organization as well. According to the current study, students’ engagement is defined as an exchange of advantages with the educational institution in the form of performing well, receiving better scores, and contributing to the institute’s leadership board. Students will produce varied influencing effects depending on their psychological expectation that the engagement would result in organizational benefits. Based on this theory, students’ engagement and disengagement could be the outcome of knowledge sharing or knowledge hiding. So, following the theory, students’ disengagement was outlined as a mediator in our research.

### Knowledge Hiding, Students’ Performance, and Students’ Disengagement

Knowledge hiding or concealing was studied as a partial counterbalance to knowledge sharing and was recognized as a sort of hoarding that should be avoided. Knowledge hiding was formally defined as an individual’s deliberate attempt to withhold or conceal information that has been sought by another person (Connelly et al., 2012). According to Connelly, there are three sorts of knowledge hiding: evasive, playing dumb, and rationalized knowledge hiding. Evasive hiding, which entails giving erroneous information or making false promises about future knowledge providing, is the first type. Playing dumb entails seeming to be unaware of the information sought by others, particularly the superiors. Rationalized hiding is the act of justifying one’s refusal to reveal information that has been sought by others (Anand et al., 2020). Replacing coworkers’ knowledge-hiding actions, counter-questioning, employees feeling insulted, plain lying, offering inaccurate information, and backstabbing are all manifestations of knowledge hiding (Anand et al., 2020; Arain et al., 2020; Irum et al., 2020). Knowledge hiding was studied as a partial counterbalance to knowledge sharing and was recognized as a sort of hoarding that should be avoided. Knowledge hiding was formally defined as an individual’s deliberate attempt to withhold or conceal information that has been sought by another person. Essentially, teaching entails the transfer of knowledge only to the degree that the stages of learning may need withholding a complicated material until the learner has mastered the particular abilities or requirements (Kiener and CRC, 2009; Karim, 2020). Overall, the adult learning principles actively argue that there is no need to conceal facts. Although the learner may not comprehend difficult material, it should not be withheld in general.

Knowledge hiding may assist the learners by giving them early access to knowledge, allowing them to play and learn *via* repeated interactions with it, and, definitely, the strategies of knowledge hiding may assist some organized or simulated learning settings, enabling for greater levels to be acquired only at important junctures. The supporting literature indicates that knowledge hiding at the teaching level, whether it is online or physical class setups, leads to several negative aspects of learning including the disengagement of the students and their performance. Class conversations are a common classroom practice in most educational systems, and they offer students with valuable learning opportunities since students and teachers cooperatively develop information and establish a shared understanding during the whole-class discussions. As a result, students must participate in such learning activities to show good performance (Abdullah et al., 2012; Mercer and Dawes, 2014). Student participation in class discussions not only includes behavioral engagement but also includes cognitive and emotional involvement (Fredricks et al., 2004). Individual students may exhibit a variety of engagement patterns, including both consistent and inconsistent combinations of student participation and cognitive and emotional engagement, as described above (Lawson and Lawson, 2013). Despite the fact that these components appear to be at different dimensions, they have significant overlap, resulting in complicated interdependencies. Regardless of the categorical imperatives and study technique issues, most scholars appear to agree that the costs of disengagement to people, families, and society increase as time goes on. As previously stated, a disengaged student has a higher chance of poor academic achievement and leaving out. There are other post-school repercussions, such as the downward spiral, which encompasses unemployment, hunger, bad health, inferior housing, and criminal justice participation. One concerning feature of this trend is that, like poverty, disengagement has a tendency to pass down over generations. Families and individuals suffer the most from disengagement, but the educational system bears the brunt of the cost. Educators who want to address the behavioral and academic consequences of disengagement frequently invest money (Baron and Corbin, 2012).

Student participation in conventional classes has been the topic of previous studies. There is a limited agreement on the definition of participation in technology-assisted learning and how it is implemented (Henrie et al., 2015). In order to learn, students must be actively involved. As the learning environment changes as a result of digitalization, it is critical to assess how student involvement is affected. It has been suggested that student involvement in online learning is misunderstood, that engagement in online learning emerges differently from participation in traditional classrooms, and that mainstream conceptualizations have overlooked how digital technologies impact engagement. Following these findings, attempts have been undertaken to construct a blended learning engagement conceptualization (Halverson, 2016). These findings are consistent with the previous engagement study, which emphasizes that context and involvement are inextricably linked (Bergdahl et al., 2020b). All these things lead to the students’ performance. In our study context, knowledge hiding at both levels of learning whether it is online or physical affects the students’ engagement in learning and refers to disengagement which could resultantly affect the students’ performance. So, we developed the following hypothesis.

*H_1_: Rationalized knowledge hiding in online teaching is associated with student’s performance.*

*H_2_. Rationalized knowledge hiding in online teaching is associated with student’s disengagement.*

*H_3_. Rationalized knowledge hiding in physical teaching is associated with student’s disengagement.*

*H_4_: Rationalized knowledge hiding in physical teaching is associated with student’s performance.*

### Mediating the Role of Student Disengagement

Academic engagement is a term that relates to a student’s level of involvement and effort in learning. A number of engagement studies have used a broad “school engagement” approach, which covers students’ social contacts with their peers among the classes as well as their involvement in after-school events, in addition to academic activities (Alrashidi et al., 2016; Wang et al., 2017). While using digital tools outside of a formal learning setting might facilitate social inclusion and support, this can have an impact on the students’ school experiences. Disengagement from education and learning can also be an outcome of the use of technology (Timmis, 2012). While student engagement is critical for learning, it is at a danger of dwindling as pupils progress through school. Disengagement, on the other hand, is more than just a lack of interest; it also involves indications like withdrawal and absenteeism, and it can entirely lead to a student dropping out (Wang and Fredricks, 2014; Tafelski et al., 2016). Despite the fact that students who are at the risk of failing or dropping out cost any educational institution time and resources, a Swedish school report found that nearly two-thirds of schools did not meet the requirements for attempting to address and attempting to prevent absences, and hardly any of the schools had explored the causative factors of student absence (Timmis, 2012).

Since it is now generally understood that disengagement raises the risk of dropping out, schools should recognize indicators of disengagement soon and take preventative efforts to reduce future absence (Skinner et al., 2008). Although, dropout is a significant problem in schools (Keyworth et al., 2019), the majority of initiatives to combat absenteeism focus on persuading the students to come to school. Observing and evaluating students’ engagement in educational activities, inactive participants, and active participants were identified as three unique student profiles by the researchers. Few of them came to the conclusion that even when the students are physically present in the classroom, they might be disengaged. In reality, many students who came to class were uninterested. Disengagement issues may worsen as a result of the implementation of digital technology (Finn and Cox, 1992). Researchers compiled the understandings of student involvement from the research literature to create a conceptualization of engagement in their key work. They claimed that participation had at least three aspects, such as behavioral, emotional, and cognitive. Lately, engagement researchers introduced a social dimension to the equation, claiming that student disengagement is a different distinct construct engagement that entails more than just a lack of involvement (Fredricks et al., 2004).

These indicators indicate that learning in a regular classroom may not conclude that learning circumstances are tied to technology. However, research on engagement and disengagement in technology-assisted learning can benefit from these findings. Researchers presented a conceptual framework that captures the cognitive and emotional dimensions of student participation in online or tech-driven learning; for example, some academics have proposed an instrument that reflects the behavioral, intellectual, and emotional elements of student involvement in tech-led learning as another way to collect and conceptualize participation in tech-led learning (Chapa, 2016). Both contributions, while useful, merely scratched the surface of the aspects of engagement and disengagement, and they were both focused on higher education (Ma et al., 2017; Halverson and Graham, 2019). In the past, there are several studies that have been conducted on knowledge sharing and its impacts on the engagement of employees, but no research has indicated the impact of knowledge hiding on students’ engagement or disengagement. So, it is necessary to design such research to identify the roles clearly. Based on the above literature about students’ disengagement which is caused by knowledge hiding in teaching and learning, and mediating a link could be explored between knowledge hiding and the outcome of students’ disengagement which is the students’ performance. In this regard, the following hypothesis were suggested.

*H_5_. Students’ disengagement mediates the relationship of the rationalized knowledge hiding in online teaching and students’ performance.*

*H_6_. Students*’ *disengagement mediates the relationship of rationalized knowledge hiding in physical teaching and students’ performance.*

**FIGURE 1:**
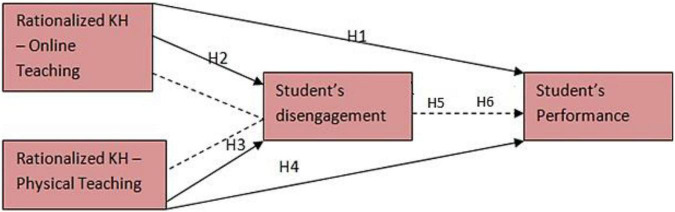
Conceptual model.

The following conceptual model (Figure 1) has been formed based on the above literature and hypothesis:

## Methodology

The present study is a cross-sectional study which is a part of the quantitative approach of research methods. It follows the hypothesis testing which falls in post-positivism where the effects of certain variables are checked on the determinant variables (Nawaz et al., 2019; Xialong et al., 2021). In this study, there are four variables in total. The independent variables are rationalized knowledge hiding (online teaching) and rationalized knowledge hiding in the physical teaching; the dependent variables are the students’ performance and the mediating variable namely students’ disengagement. The population of the study is the enrolled students in the universities of China. The sampling method used in this study is convenience sampling because the students were reached based on the ease of approaching the sample (Avotra et al., 2021; Yingfei et al., 2021). The data were collected based on the consent of the respondents and they were approached with prior appointments through coordinators of their degree programs to avoid inconvenience through the process. The hypothesis formulated in the study is accepted/rejected based on the statistics obtained from the structural equation modeling (SEM) using the software, SmartPLS Version 3.

### Instrument

The instrument used in the survey study is a Questionnaire. The Questionnaire has been developed on the 5-point Likert scale where the degrees of responses ranged from 1 to 5, where 1 is coded as strongly disagree and the response, 5 is coded as strongly agree. The instrument was adapted by gathering the scales for respective variables used in the past; however, the first part of the Questionnaire contained the demographic details of the respondents. The scales for the rationalized knowledge hiding for online and physical teaching methodologies had been measured through a 4-item scale adapted from Connelly et al. (2012). The sample items for the rationalized knowledge hiding for online teaching include, “In online class, the teacher explained that the information is confidential and only available to people on a particular project,” and “In online class, the teacher remained silent”. While, the sample items for rationalized knowledge hiding for physical teaching include “In physical class, the teacher does not allow me to share this knowledge/information with others” and “In physical class, the teacher explained that he could not provide information,” while these items were modified accordingly. The variable, students’ disengagement was taken from the study by Bergdahl et al. (2020b); it consisted of 6-items that address the disengagement measuring scale for the students. Sample items for disengagement include, “We, the students, are often more knowledgeable about it than the teacher, and therefore left to decide how technologies should be used for learning.” And, “As we are merely instructed to sit alone and search the Internet, I would rather do the school assignments at home than in the classroom.” Furthermore, the students’ performance was measured *via* a 5-item scale that was used by Neroni et al. (2019). The sample items for students’ performance include, “I often find myself questioning things I hear or read in a course to decide if I find them convincing.”

### Demographic Details

The data collection was done through survey methods where questionnaires were distributed to the respondents and the collected data was assembled and analyzed. The responses obtained for the demographic questions were analyzed using the frequencies and the percentages. The results of the demographic details can be seen in Table 1. The demographic sheet consisted of questions, such as age, gender, education, and the department of the respondents (Baig et al., 2014). There was a total of 246 respondents who were students, both male and female enrolled in different degrees. There were 131 men and 115 women among the total respondents. The highest frequency among the different age brackets was those between 15 and 20 who were 130, while 70 were between the ages of 21 and 25, and the least was 46 among the ages, 26–30. No respondent was found whose age is above 31. Moving to the educational level, the highest frequency was found for the master’s students that were 121, followed by the bachelor’s and then Ph.D., and others. However, the highest frequency in the respondents was of natural sciences students, followed by management sciences than the social sciences and fine arts.

**TABLE 1:** Demographic analysis.

Demographics	Frequency	Percentage
**Gender**		
Male	131	53.25%
Female	115	46.75%
**Age**		
15–20	130	53.20%
21–25	70	28.45%
26–30	46	18.35%
31 and above	–	–
**Education level**		
Bachelors	96	39.02%
Masters	121	49.20%
Ph.D. and others	29	11.78%
**Department**		
Natural Sciences	82	33.33%
Social Sciences	58	23.59%
Management Sciences	71	28.86%
Fine Arts	35	14.22%

*N = 246.*

## Data Analysis and Results

The data analysis was done in the current study using the software, SmartPLS version 3. It is used to carry out the partial least square SEM. In this approach, the data are analyzed simultaneously for all the relationships mentioned in the conceptual model. The advantage of this analytical technique is that a variable used as a dependent variable in one relationship can be used as an independent variable for the next relationship (Henseler et al., 2015). The Questionnaire consisted of 19 items in total. Though the scale has already been used, it was verified for the present data through Cronbach’s alpha, discriminant, and convergent validity.

### Measurement Model

**FIGURE 2:**
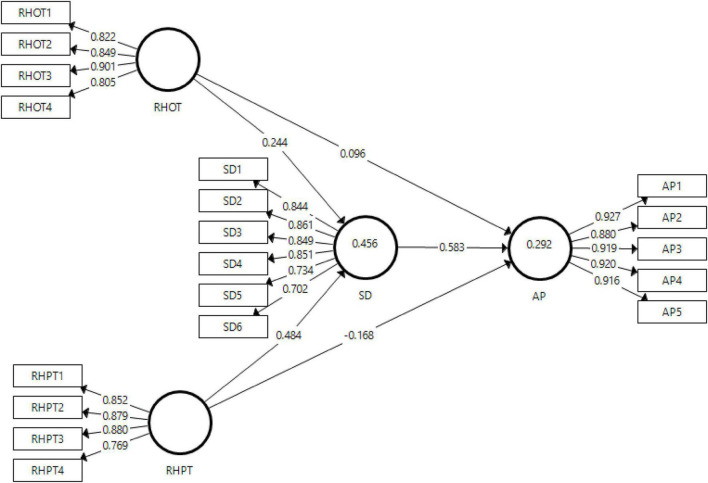
The output of the measurement model. RHOT, rationalized knowledge hiding—online education; RHPT, rationalized knowledge hiding—physical education; SD, students’ disengagement; AP, students’ performance.

The reliabilities used in this study are Cronbach’s reliability and composite reliabilities. However, the discriminant validity has been checked *via* heterotrait-monotrait ratio (HTMT) and Fornell-Larcker Criteria. The convergent validity has been checked through the factor loadings and the average variances extracted (AVE). The output obtained from the measurement model has been presented in Figure 2.

The reliabilities used in this study are Cronbach’s alpha and composite reliability. The convergent validity has been measured through the factor loadings and the average variances extracted. For the variables to be valid, the criteria set in the literature is 0.6, for the reliabilities threshold, it is 0.7 and for AVE, it is 0.5, such that all the values should be above this cut-off (Baig and Waheed, 2016; Abdalla et al., 2021). All the values shown for factor loadings are all above the threshold of 0.6 which shows the convergent validity for all the variables. Similarly, the values for alpha reliabilities are above 0.8 which are satisfactorily above the cut-off values showing that the data obtained is reliable. Furthermore, the AVE statistics for the dependent, independent, and mediating variables are above 0.6 which confirms the convergent validity. The results for all tests are listed in Table 2.

**TABLE 2:** Factor loadings and reliabilities.

Variables	Factor loadings		VAF	Cronbach’s alpha	Composite reliability	AVE
Academic performance	EC1	0.927	4.402	0.950	0.961	0.833
	EC2	0.880	3.031			
	EC3	0.919	4.464			
	EC4	0.920	4.413			
	EC5	0.916	4.018			
Student disengagement	SD1	0.844	2.544	0.893	0.919	0.655
	SD2	0.861	2.846			
	SD3	0.849	2.661			
	SD4	0.851	2.602			
	SD5	0.734	1.783			
	SD6	0.702	1.677			
Rationalized hiding – online teaching	OT1	0.822	2.107	0.866	0.909	0.714
	OT2	0.849	2.018			
	OT3	0.901	3.074			
	OT4	0.805	2.131			
Rationalized hiding – physical teaching	PT1	0.852	2.185	0.867	0.909	0.716
	PT2	0.879	2.428			
	PT3	0.880	2.453			
	PT4	0.769	1.740			

*N = 246.*

The discriminant validity in the present study has been checked through the HTMT ratio and the Fornell-Larcker criterion. The acceptance criterion for the HTMT ratio is that it should be below 0.8 and for Fornell-Larker, all the highest values of each column should lay on the top. Table 3 shows the HTMT ratio of the data for this study and all the values are below the maximum threshold. Similarly, the results of the Fornell-Larcker criteria are also significant showing the highest value of each column at the top. The results of the Fornell-Larcker criteria are listed in Table 4.

**TABLE 3:** Heterotrait-monotrait ratio (HTMT) ratio.

	AP	RHOT	RHPT	SD
AP				
RHOT	0.345			
RHPT	0.300	0.783		
SD	0.572	0.656	0.734	

*RHOT, rationalized knowledge hiding—online education; RHPT, rationalized knowledge hiding—physical education; SD, students’ disengagement; AP, students’ performance.*

**TABLE 4:** Fornell-Larcker criteria.

	AP	RHOT	RHPT	SD
AP	0.913			
RHOT	0.317	0.845		
RHPT	0.278	0.688	0.846	
SD	0.529	0.577	0.652	0.809

*RHOT, rationalized knowledge hiding—online education; RHPT, rationalized knowledge hiding—physical education; SD, students’ disengagement; AP, students’ performance.*

Furthermore, the r-square values obtained from the variable, students’ disengagement is 45.6% and for students’ performance, it is 29.2% which shows a significant prediction of the model. The reference values for *f*-square are such that the values equal to or more than 0.02 show a small effect, values equal to or more than 0.15 show a medium effect, while the values equal to or more than 0.35 show a large effect. These values have been reported with direct effects of the study (Selya et al., 2012).

### Structural Model

**FIGURE 3:**
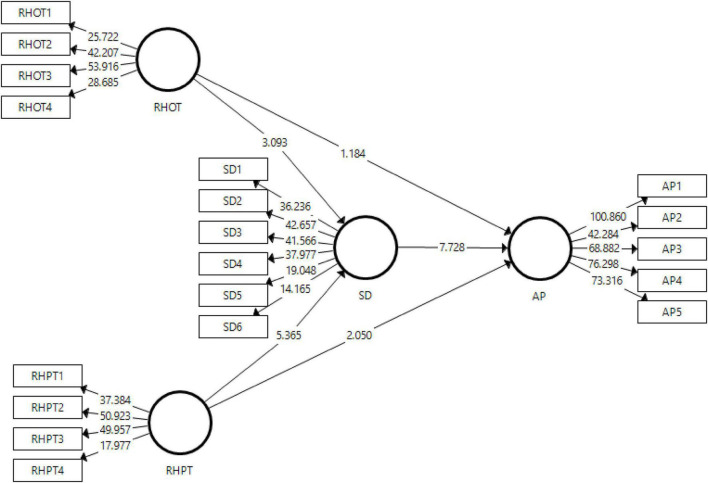
The output of the structural model. RHOT, rationalized knowledge hiding—online education; RHPT, rationalized knowledge hiding—physical education; SD, students’ disengagement; AP, students’ performance.

Output for the third stage of SEM has been shown in Figure 3 which presents the structural model. Following the results produced from the software, the hypothesis is either accepted or rejected. The output algorithm for the structural model measurement is shown in Figure 3.

The statistics used as the criteria for the acceptance of the hypothesis are *p*-values, *t*-values, *r*-square, original sample means, and SDs. The results obtained for the hypothesis acceptance criteria are mentioned in Table 5. The direct effects of the variables have been checked and given in the table. The hypothesis for the association of rationalized knowledge hiding and online thinking has been rejected to play any role in student performance (*t*-stats = 1.184, *p* > 0.05). The second hypothesis (H_2_: rationalized knowledge hiding in online teaching is associated with students’ disengagement) has been accepted with *t*-stats = 3.093 and *p* < 0.05. The third hypothesis (H_3_: rationalized knowledge hiding in physical teaching is associated with students’ disengagement) is accepted with *t*-stats = 2.050 and *p* < 0.05; however, it shows the negative effect of rationalized knowledge hiding—physical teaching on the students’ disengagement. Similarly, the fourth hypothesis (H_4_: Rationalized knowledge hiding in physical teaching has an association with students’ performance) is accepted with *t*-stats = 5.365.

**TABLE 5:** Direct effects.

Paths	H	O	*M*	SD	*f*-square	*T*-Statistic	*P*-value	Results
RHOT → SP	H_1_	0.096	0.094	0.081	0.008	1.184	0.237	Rejected
RHOT → SD	H_2_	0.244	0.246	0.079	0.056	3.093	0.002**	Accepted
RHPT → SD	H_3_	0.484	0.484	0.090	0.227	5.365	0.000***	Accepted
RHPT → SP	H_4_	−0.168	−0.164	0.082	0.017	2.050	0.041*	Accepted

***p < 0.001, **p < 0.005, *p < 0.05.

*H, hypothesis; O, original sample; M, sample mean; SD, standard deviation; RHOT, rationalized knowledge hiding—online education; RHPT, rationalized knowledge hiding—physical education; SD, students’ disengagement; AP, students’ performance.*

The indirect effects of the study are mentioned in Table 6. The first relationship for the mediation of students’ disengagement in the relationship of rationalized knowledge hiding—online teaching and students’ performance has been accepted with *t*-stats = 2.995. In addition, the other mediation of the students’ disengagement between the relationship of rationalized knowledge hiding—physical teaching and students’ performance is also accepted with *t*-stats = 4.338. Both hypotheses for mediation showed partial mediation as the value of variance accounted for (VAF) under 20% shows no mediation, between 20 and 80% shows partial mediation and above 80% shows full mediation (Bari et al., 2020).

**TABLE 6:** Indirect effects.

Paths	H	O	*M*	SD	*T*-Statistic	VAF	*P*-value	Results
RHOT → SD → SP	H_5_	0.142	0.141	0.047	2.995	59.7%	0.003	Partial Mediation
RHPT → SD → SP	H_6_	0.282	0.278	0.065	4.338	62.6%	0.000	Partial Mediation

*H, hypothesis; O, original sample; M, sample mean; SD, standard deviation; VAF, variance accounted for; RHOT, rationalized knowledge hiding—online education; RHPT, rationalized knowledge hiding—physical education; SD, students’ disengagement; AP, students’ performance.*

## Discussion

This research was conducted following the research gap found in previous similar studies in which the role of knowledge hiding was a little bit analyzed in the educational context. A lot of work has been done in the past on the knowledge hiding concept regarding the organizational setups. This provided us the opportunity to find the impact of knowledge hiding in the context of education learning. As described by Connelly et al. (2012), knowledge hiding was divided into three categories which referred to the concept. Among these three, we tried to explore the role of rational or justified knowledge hiding in our study context. In the case of teaching, the rationalized hiding provided the basis for the significance of provided justifications. In this approach, teachers or tutors had the logic and the justification for the concealment of knowledge. We got mixed results on identifying the impacts of rationalized knowledge hiding among physical education students. There was a gap in the research for the impact of such knowledge hiding on students’ disengagement. Very few studies in the past were carried out on the impact of knowledge sharing regarding students’ engagement. This aspect was also leading toward the students’ performance in terms of learning. A lot of work has been carried out on the impact of knowledge sharing, engaging the students in knowledge dissemination and their academic achievements. But there was a scope of finding out the impact of rationalized knowledge hiding on students’ achievements or academic performance. This yielded a significant contribution in the educational field. It also provided the scope of strategies or policies in the performance management of the higher education sector and the students’ exchange of knowledge.

Direct and indirect variables along with mediation were hypothesized and analyzed in our research. Our first hypothesis which was about the relationship of rationalized knowledge hiding in online teaching and its association with students’ performance was rejected, indicating that there was no role of rationalized knowledge hiding in online teaching with regard to the students’ performance. The second hypothesis was about checking the impact of rationalized knowledge hiding in the online medium of students’ disengagement and it was accepted showing that students could easily disengage in online teaching setups due to different distractions. A few research has been conducted in the past indicating the role of digital technologies in the disengagement of students toward learning which are in line with our obtained results (Bergdahl et al., 2020b). The third hypothesis which was about the role of rationalized knowledge hiding on students’ disengagement in physical learning was accepted showing the significance of the relationship. It meant that such a kind of knowledge hiding could yield students’ disengagement due to the fact that the teacher is in front of the class. When students ask for any information, either they get false information or the knowledge is hidden in any other ways of knowledge hiding. This thing leads to students’ disengagement in response. The fourth hypothesis indicated that the rationalized knowledge hiding in the physical medium of teaching had a positive association with students’ performance. These results proved that some of the previous studies were justified.

Some researchers like Garg et al. (2021), proposed that rationalized knowledge hiding had a significant positive impact on the students’ performance. The analogy between our results and this research could be drawn easily. The third assumption of our study was about students’ disengagement and its impact on their performance. This also got accepted and proved the results of many previous researchers who stated that students’ disengagement could lead to their negative performance, such as low grades and the ultimate drop out. Some researchers like Chipchase et al. (2017), Bergdahl et al. (2020b), and Schnitzler et al. (2021) were of the same views. Our last two hypotheses were about the mediating role of student disengagement in online and physical teaching between knowledge hiding and students’ performance. In contrast to the results of Hypothesis 1, our second last hypothesis about online learning rationalized knowledge hiding, was accepted due to the fact that disengagement was associated with the rationalized knowledge hiding and it could develop a mediating link between the knowledge hiding and the students’ performance. Our last hypothesis which was about the mediating link of student disengagement between rationalized knowledge hiding in physical learning and students’ performance was proved significant as well. The mediating role of student disengagement had not been studied before but its counter-part which is student engagement, has been studied for its mediating role in many studies (Chapa, 2016; Siddiqi, 2018; García-Martínez et al., 2021).

## Theoretical Contributions

The study has certain theoretical contributions: (1) First of all, no prior study has attempted to check the impact of rationalized knowledge hiding from teachers on the students’ performance. (2) Second, the knowledge hiding behavior of the teachers has been found as an important factor in affecting the students’ performance in their educational roles. (3) Third, this will be an important breakthrough proposing an adequate check and balance and in the monitoring of the teaching classes to ensure that no knowledge hiding is exhibited, be it rationalized.

## Limitations and Future Research

Although the present study has contributed to the literature, still there are some limitations to the present study that gives an opportunity for future research. For example, (1) It has a sample size of 246 which is a small sample concerning the population of the students. Future studies should be conducted with a large sample size and probability sampling to ensure rigor in the study. (2) Future studies should be conducted considering the role of online classes at different times *t* to understand if online classes make any difference from physical teaching. (3) This study is conducted in Chinese universities; it should be conducted in other countries of the world to ensure the generalization of the results. (4) This study can be replicated with more mediating variables (like understanding of content, interest, lifestyle, etc.) and moderating variables (gender, technical support, social networking, etc.). Furthermore, the present study has measured the effect of behaviors on performance; however, the perceived perceptions and self-efficacy are important factors that mold the outcome of the behavior. Therefore, it is advised to check the moderating effects of these variables in future studies.

## Practical Implications

This study has many implications in the real world. For example, this study will be helpful for the governing body of the universities in warranting the maximum quality outcome of the teaching classes, workshops, and seminars conducted either physically or online so that no instructor involves in rationalized knowledge hiding. Organizations, especially universities, can make sure of the maximum sharing of knowledge by being involved in positive reinforcement behaviors to motivate the instructors. Third, the organizations can devise such teaching content that involves an active listening pattern of the lectures delivered, so that the students are kept engaged in the lessons. Furthermore, this study has provided empirical evidence and deep understanding and suggestions for the teachers, instructors, and academic administrators to provide such a culture among the students and teachers that help them in achieving the desired shared goals by reducing the undesirable behaviors.

## Conclusion

Students’ performance has been a key marker for the success of nations and the quality of educational institutes. Keeping students engaged with their lessons has been the focus of researchers for many years. The post-pandemic scenario has changed all the parameters in education that had been prevailing lately, especially after the online teaching schedules. There had been many studies exploring the engagement and disengagements of the students in their curriculum and education-related activities and lessons. The present study has also assessed the role of rationalized knowledge hiding in the online and physical classes exhibited on behalf of the teacher, in shaping the disengagement of students from their lessons and their final effects on the students’ performances. Rationalized teaching in online teaching has not been found to have any role in the students’ performance; however, it has shown a significant relationship with the students’ disengagement. However, the results of the study show that when the students experience rationalized knowledge hiding in physical teaching, it leads to the students’ disengagement and causes a decline in the students’ overall performance. Furthermore, the mediating role of students’ disengagement has been found significant in this study. When the students experience rationalized knowledge hiding from the teachers, they disengage from the studies and it affects their performances.

## Data Availability Statement

The original contributions presented in the study are included in the article/supplementary material, further inquiries can be directed to the corresponding author/s.

## Ethics Statement

The studies involving human participants were reviewed and approved by the Jiangnan University, China. The patients/participants provided their written informed consent to participate in this study. The study was conducted in accordance with the Declaration of Helsinki.

## Author Contributions

QX conceived and designed the concept, and wrote the manuscript. YJ collected the data and provided technical support. Both authors have read and agreed to the published version of the manuscript.

## Conflict of Interest

The authors declare that the research was conducted in the absence of any commercial or financial relationships that could be construed as a potential conflict of interest.

## Publisher’s Note

All claims expressed in this article are solely those of the authors and do not necessarily represent those of their affiliated organizations, or those of the publisher, the editors and the reviewers. Any product that may be evaluated in this article, or claim that may be made by its manufacturer, is not guaranteed or endorsed by the publisher.

## Appendix

**Table:** Questionnaire.

S No.	Items
***Academic performance*** (Neroni et al., 2019)	
1	When reading for a course, I make up questions to help focus my reading
2	I often find myself questioning things I hear or read in a course to decide if I find them convincing
3	When a theory, interpretation, or conclusion is presented in a course or in the readings, I try to decide if there is good supporting evidence
4	I treat the course material as a starting point and try to develop my own ideas about it
5	Whenever I read or hear an assertion or conclusion in a course, I think about the possible alternatives
***Students’ disengagement*** (Bergdahl et al., 2020a)	
1	I have to learn how applications/computer programs work for myself
2	We, the students, are often more knowledgeable about it than the teacher, and therefore left to decide how technologies should be used for learning
3	As we are merely instructed to sit alone and search the Internet, I would rather do the school assignments at home than in the classroom
4	I am often encouraged to find knowledge by “searching the internet” and I want the lesson to be more thought-through (deliberate) than that
5	It makes me feel upset/resigned that the same students repeatedly contribute less to the group work, even though we have computers and can easily share the work
6	My engagement decreases when working alone on the computers/tablets
***Rationalized knowledge hiding—online teaching*** (Connelly et al., 2012)	
1	In the online class, the teacher does not allow me to share this knowledge/information with others
2	In the online class, the teacher explained that the information is confidential and only available to people on a particular project
3	In the online class, the teacher explained that, I could not provide information
4	In the online class, the teacher remained silent
***Rationalized knowledge hiding—physical teaching*** (Connelly et al., 2012)	
1	In the physical class, the teacher does not allow me to share this knowledge/information with others
2	In the physical class, the teacher explained that the information is confidential and only available to the people on a particular project
3	In the physical class, the teacher explained that I could not provide information
4	In the physical class, the teacher remained silent
